# Current epidemiological evidence for predisposition to high or low intensity human helminth infection: a systematic review

**DOI:** 10.1186/s13071-018-2656-4

**Published:** 2018-01-31

**Authors:** James E. Wright, Marleen Werkman, Julia C. Dunn, Roy M. Anderson

**Affiliations:** 1 Department of Infectious Disease Epidemiology, Imperial College London, St. Mary’s Campus, London, W2 1PG UK; 2 London Centre for Neglected Tropical Disease Research (LCNTDR), Department of Infectious Disease Epidemiology, Imperial College London, St Mary’s Campus, London, W2 1PG UK; 3 The DeWorm3 Project, The Natural History Museum of London, London, SW7 5BD UK

**Keywords:** Predisposition, Helminths, Systematic review, *Ascaris*, *Trichuris*, Hookworm, Schistosomiasis

## Abstract

**Background:**

The human helminth infections include ascariasis, trichuriasis, hookworm infections, schistosomiasis, lymphatic filariasis (LF) and onchocerciasis. It is estimated that almost 2 billion people worldwide are infected with helminths. Whilst the WHO treatment guidelines for helminth infections are mostly aimed at controlling morbidity, there has been a recent shift with some countries moving towards goals of disease elimination through mass drug administration, especially for LF and onchocerciasis. However, as prevalence is driven lower, treating entire populations may no longer be the most efficient or cost-effective strategy. Instead, it may be beneficial to identify individuals or demographic groups who are persistently infected, often termed as being “predisposed” to infection, and target treatment at them.

**Methods:**

The authors searched Embase, MEDLINE, Global Health, and Web of Science for all English language, human-based papers investigating predisposition to helminth infections published up to October 31st, 2017. The varying definitions used to describe predisposition, and the statistical tests used to determine its presence, are summarised. Evidence for predisposition is presented, stratified by helminth species, and risk factors for predisposition to infection are identified and discussed.

**Results:**

In total, 43 papers were identified, summarising results from 34 different studies in 23 countries. Consistent evidence of predisposition to infection with certain species of human helminth was identified. Children were regularly found to experience greater predisposition to *Ascaris lumbricoides*, *Schistosoma mansoni* and *S. haematobium* than adults. Females were found to be more predisposed to *A. lumbricoides* infection than were males. Household clustering of infection was identified for *A. lumbricoides*, *T. trichiura* and *S. japonicum*. *Ascaris lumbricoides* and *T. trichiura* also showed evidence of familial predisposition. Whilst strong evidence for predisposition to hookworm infection was identified, findings with regards to which groups were affected were considerably more varied than for other helminth species.

**Conclusion:**

This review has found consistent evidence of predisposition to heavy (and light) infection for certain human helminth species. However, further research is needed to identify reasons for the reported differences between demographic groups. Molecular epidemiological methods associated with whole genome sequencing to determine ‘who infects whom’ may shed more light on the factors generating predisposition.

**Electronic supplementary material:**

The online version of this article (10.1186/s13071-018-2656-4) contains supplementary material, which is available to authorized users.

## Background

The human helminths are a group of parasites whose impact is felt throughout the world by the poorest individuals within the poorest communities. There are two predominant helminth sub-groups, nematodes (or roundworms) and trematodes (or flukes). The former group includes soil-transmitted helminths (STH), i.e. *Ascaris lumbricoides*, *Trichuris trichiura*, *Necator americanus* and *Ancylostoma duodenale* as well as the filarial worms, which cause lymphatic filariasis (LF) and onchocerciasis. Meanwhile, the latter group consists of three species causing schistosomiasis (*Schistosoma mansoni*, *Schistosoma haematobium* and *Schistosoma japonicum*).

Global estimates suggest that approximately 1.5 billion people are infected with at least one intestinal nematode [1]. Similarly, the number of individuals infected with schistosomiasis, LF and onchocerciasis worldwide are estimated to be 250 million, 36 million and 30 million, respectively [2]. Whilst not generally considered fatal, helminth infections are strongly associated with severe morbidity, especially in children [3]. Associations between chronic helminth infection and various non-communicable diseases such as bladder cancer (*S. haematobium*), anaemia (hookworm), and asthma (*A. lumbricoides*) have also been identified [4]. Cumulatively, helminth infections result in approximately 12 million disability-adjusted life years (DALYs) worldwide [2], with the majority (5.18 million) attributed to STH [1]. The greatest geographical burden of infection is found in sub-Saharan Africa and South-East Asia [2].

Whilst the World Health Organisation (WHO) treatment guidelines vary between helminth species, most are currently tailored towards morbidity control (although elimination programs are in operation for the filarial infections). However, there has been a recent shift in focus since the 2012 London Declaration, in which pharmaceutical companies, donors, endemic countries, and non-governmental organisations (NGOs) committed to target the control, elimination, or eradication of ten Neglected Tropical Diseases (NTDs) by 2020 [5]. The target for LF is global elimination, whilst for onchocerciasis and schistosomiasis, elimination is targeted in selected African countries as well as in Latin America (onchocerciasis) and the Western Pacific Region (schistosomiasis). For STH, the aim is to regularly treat 75% of pre-school-aged children (pre-SAC) and school-aged children (SAC) in need of treatment and to achieve 75% treatment coverage in these two groups in all endemic countries [6].

Given these 2020 targets, some countries have been moving from treatment regimens aimed at morbidity control to ones focussing on transmission elimination. This has largely been seen through a shift away from school-based deworming programs, and towards mass drug administration (MDA) campaigns, in which entire communities are treated.

However, as successive rounds of MDA move the prevalence of infection to lower levels, continuing to treat entire populations may not remain the most efficient, nor most cost-effective, control strategy. Instead, it may be more pertinent to identify individuals or groups of people with defined characteristics who are consistently infected, despite receiving repeated rounds of treatment, and target deworming efforts at them [7]. These individuals can be described as “predisposed” to infection. Predisposed individuals are also likely to continue re-introducing infectious material into the local environment, perpetuating transmission and increasing the incidence amongst those who had successfully cleared the infection via prior treatment.

A previous review into predisposition to helminth infections was published in 1990 [8]. Since 1990, however, multiple studies have been conducted that address this issue. Therefore, the present review is intended as an update. With the London Declaration shifting attention firmly towards elimination of helminth transmission, the notion of identifying persistently infected individuals is becoming increasingly relevant. Hence, in this study, a systematic literature review is performed to investigate the current evidence for predisposition.

This review aims to outline the various definitions for predisposition used in the literature and the different methods for assessing it. Current understanding with respect to predisposition to human helminth infection and possible causative factors will be summarised, and gaps in present knowledge and areas for further work will be identified.

## Methods

This systematic review was conducted in accordance with the Preferred Reporting Items for Systematic Reviews and Meta-Analyses (PRISMA) guidelines, the completed checklist for which is in Additional file 1: Table S1.

### Search strategy

All data analysis studies reported in the literature on the topic of predisposition to human helminth infection were included, with the intention of identifying longitudinal studies in which the same individuals had their infection status measured at multiple time points. All studies focused on helminths in humans, with no publication date limitations. Furthermore, the search was not limited to certain geographical regions.

The authors searched Embase, MEDLINE, Global Health, and Web of Science for all papers published up to October 31st, 2017. Search terms, derived from three general sections (i.e. the disease, the population of interest, and the topic of interest), were ((“soil-transmitted helminths” OR “soil transmitted helminths” OR geohelminths OR helminth* OR hookworm OR *A. lumbricoides* OR ascariasis OR *T. trichiura* OR trichuriasis OR “Ascaris lumbricoides” OR “Trichuris trichiura” OR “*Necator americanus*” OR “*Ancylostoma duodenale*” OR schistosomiasis OR *Schistosoma*? OR Bilharzia OR LF OR “Lymphatic Filariasis” OR Onchocerciasis) AND (Human* OR adults OR children OR “school-aged children” OR SAC OR “pre-school-aged children” OR pre-SAC) AND (predisposition OR clustering OR propensity OR reinfection OR aggregation OR susceptibility OR clumping OR heterogeneity OR non-uniform OR random)). Citations for all identified papers were imported into Endnote X7 (Thomson Reuters, New York, USA).

### Selection criteria

Abstracts and titles were reviewed for all identified papers, with those deemed unsuitable discarded. Inclusion criteria for this initial decision were purposefully broad to increase the chances of identifying all relevant papers. However, the papers included had to be summarising results from human-based epidemiological studies, be written in English, and have a longitudinal component whereby follow-up post-treatment enabled individual-level comparisons in pre- and post-treatment infection levels. The included papers were analysed in detail, but with two additional inclusion criteria. Firstly, the full text had to be available, else it could not be reviewed. Secondly, studies had to have successfully enrolled at least 10 people testing positive for helminth infection over multiple time points to allow for sufficient power in any statistical analysis undertaken. This review excluded previously published reviews on the epidemiology of human helminth infection as these papers did not contain any new data. However, these papers were read to identify any references not yet included in the study.

### Data extraction

Relevant information from the selected articles was extracted and entered manually into a standardized Excel datasheet. For each full-text included, the following information was extracted: title, author(s), year of publication, study population/country/region, study design, helminth species, number of subjects followed longitudinally, diagnostic test(s) used, number of diagnostic slides used (if applicable), duration of follow up, definition of predisposition used, presence of predisposition (Yes/No), the statistical test used to determine predisposition, evidence for predisposition (statistical result), method used to identify risk factors, risk factors identified, and potential causes of bias.

### Data synthesis

Summary data are presented on the geographical areas in which studies have been conducted, the years in which papers were published, the varying definitions used to describe predisposition, and the statistical tests used to determine its presence. The evidence for predisposition is then presented, stratified by helminth species. Finally, potential risk factors of predisposition identified in the papers are summarised.

## Results

### Summary of papers

**Fig. 1:**
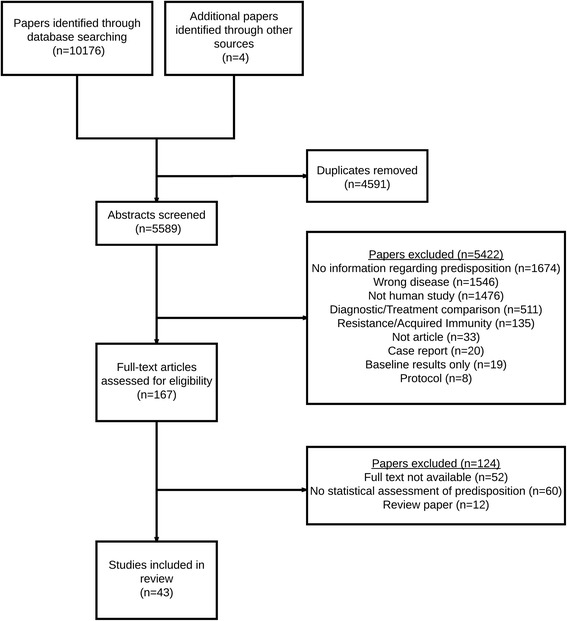
PRISMA diagram summarising inclusion and exclusion of all identified papers

The search strategy yielded 10,176 papers across the four databases, with four papers added from previous reviews. After removing duplicates, 5589 titles and abstracts were screened. Based on the first set of inclusion criteria, 5422 papers were excluded, and 167 papers were assessed for eligibility. Of those eligible, 52 did not have a full text available, 12 were previous reviews and 60 were excluded based on inclusion criteria. Hence, a total of 43 papers, presenting results from 34 different studies, were included in this review for the qualitative synthesis of the published papers (Fig. 1).

**Fig. 2:**
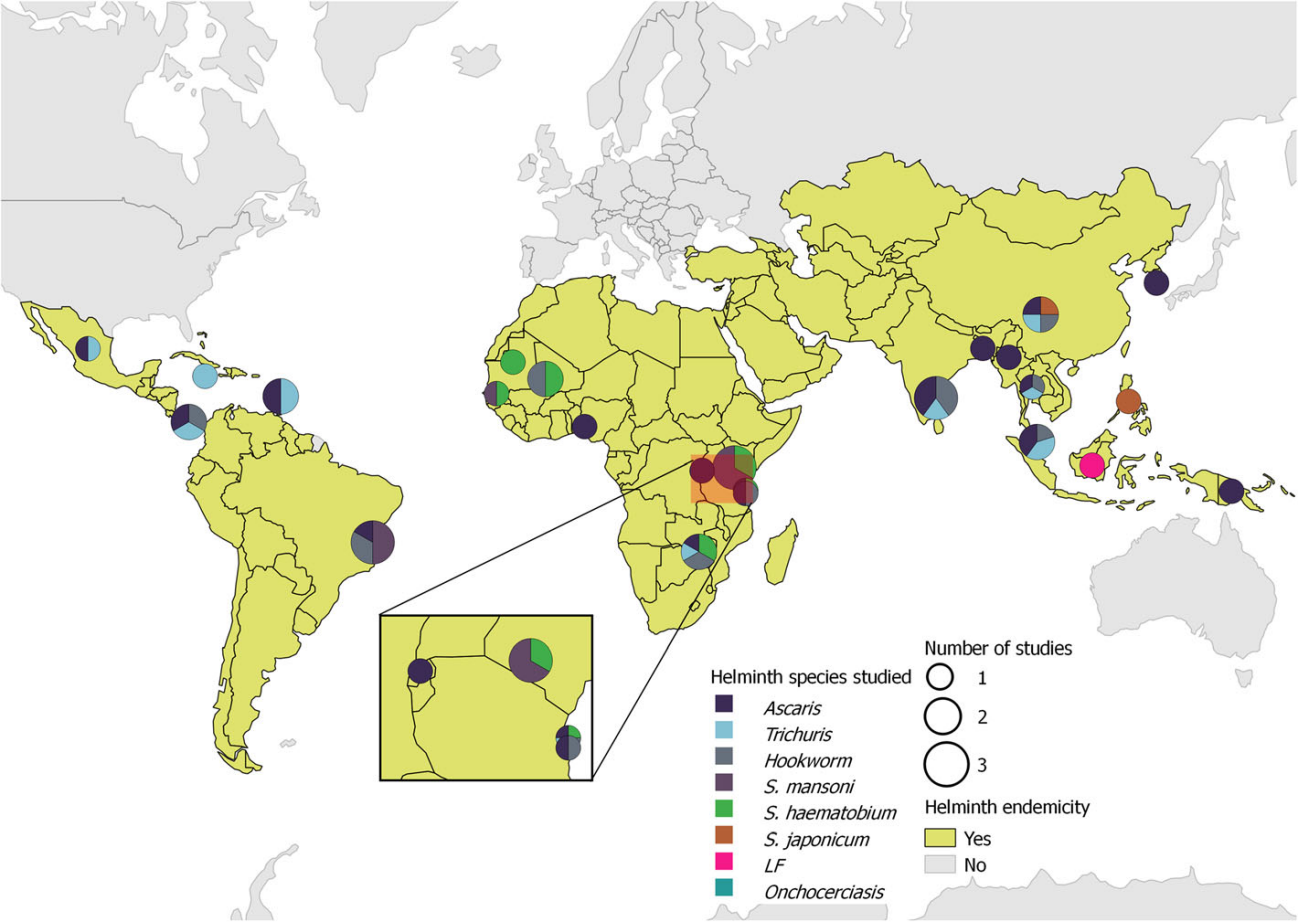
Map showing the geographical distribution of all included papers (*n* = 43). The size of each circle is proportional to the total number of studies conducted in each country. The sections of each circle represent each helminth species investigated in that country

**Fig. 3:**
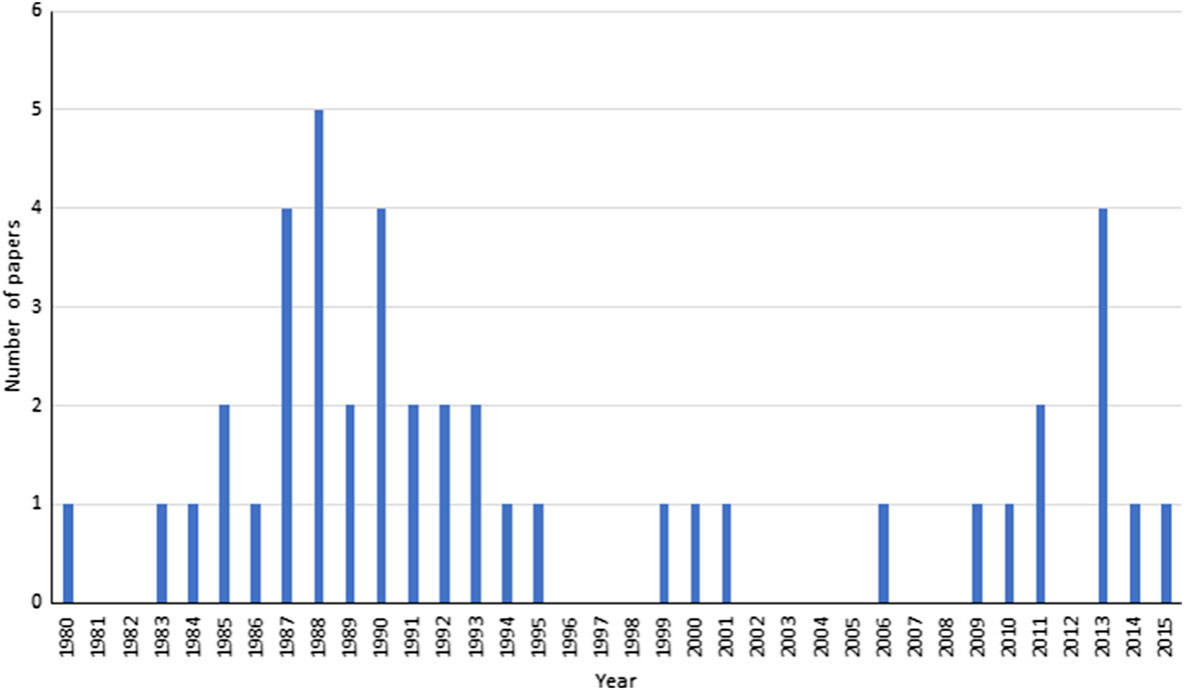
Distribution of publication year for the included papers (*n* = 43)

Studies were identified from a total of 23 countries, with the majority undertaken in sub-Saharan Africa (13 papers, 30.2%) and Asia (12 papers, 27.9%) (Fig. 2). Identified papers were published between the years 1980 and 2015, with most published in the late 1980s and early 1990s, proceeded by less research in the topic over subsequent years. However, there has been an increase in published papers in the last five years (Fig. 3). Participants were selected from whole communities for 21 studies (61.8%), whilst pre-SAC and SAC were the primary focus of two (5.9%) and 11 (32.4%) studies, respectively. Given that only two studies summarised results from pre-SAC, these studies were combined with those focussing on SAC, thus forming a “Child” group for the stratification of results.

*Ascaris lumbricoides* was the most commonly studied helminth (22 papers, 51.2%) followed by hookworm (17 papers, 39.5%) and *T. trichiura* (15 papers, 34.9%). Fourteen papers presented results on schistosome infections, of which six reported *S. mansoni,* five reported *S. haematobium* and three reported *S. japonicum*. No papers were identified which reported longitudinal results for LF or onchocerciasis. In general, species determination between *N. americanus* and *A. duodenale* was not conducted. As such, results will be presented under the single group entitled “hookworm”.

### Definition of predisposition

Considerable inconsistency was observed in the definition of predisposition used in the literature, with the 43 papers employing a total of 15 different definitions (Table 1). The most frequently used definition was the idea of participants being consistently infected at multiple time points (11 papers, 25.6%). A further nine papers (20.9%) modified this slightly to expressly define predisposition as being consistently infected with *high-intensity* helminth infection over multiple time points, whilst two (4.7%) considered predisposition as being infected with infections of the *same intensity* over multiple time points.

**Table 1:** Frequency of definitions for predisposition utilised within the included papers

Definition of predisposition	No. of papers^a^
Consistently infected at multiple time points	11
Consistent high intensity infections at multiple time points	9
Pre-treatment infection status as predictor for future infection	6
Pre-treatment intensity of infection amongst those infected post-treatment	4
Clustering of high-intensity infections within households	3
Consistent high intensity infections amongst family members	2
Consistently infected at same intensity level across multiple time points	2
Association between pre-treatment and post-treatment worm burdens	1
Clustering of infections within households	1
Consistently high intensity infections within families at multiple time points	1
Correlation between pre-treatment infection/intensity and reinfection intensity	1
Familial aggregation of re-infection	1
Individual predisposition	1
Ratio of proportion predicted to be infected and observed proportion	1
Trend in reinfection with increasing pre-treatment intensity	1
Whether those heavily infected at baseline were more likely to be infected at follow-up	1

^a^Total may exceed 43 due to some papers using multiple definitions

The idea of within household clustering of helminth infection was used as a definition of predisposition by four papers. Of which, three (7.0%) focused on the clustering of high-intensity infections and one (2.3%) identified infections of any intensity.

### Statistical methods used

As with the definitions of predisposition, there was considerable variability in the statistical methods used to assess its presence (Table 2). The most common technique used was a rank correlation coefficient; 13 papers (30.2%) used Kendall’s tau, and eight (18.6%) used Spearman’s rank coefficient. These techniques, which were conducted on either egg counts (EPG) or worm burdens (via expulsion chemotherapy), rank individuals according to their raw counts at each time point. Pairwise comparisons in these rankings are evaluated across multiple time points. In each case, small *P*-values from the rank tests were indicative of those with high (or low) egg counts (or worm burdens) at the first time point having the same infection intensity (relative to the rest of the sample when ranked) at the second time point. Thus, these individuals were predisposed to either high or low infection intensities. The use of rank correlation coefficients was largely employed in papers defining predisposition as being consistently infected over multiple time points, despite rounds of treatment and periods of reinfection, relative to others in the sample. Since it is expected that mean intensity falls after multiple rounds of treatment, relativity is important in ranking statistical tests. It is an individuals’ ranking relative to others that indicates predisposition, not the absolute value of infection intensity.

**Table 2:** Frequency of statistical tests used to determine presence of predisposition within the included papers

Statistical test used	No. of papers^a^
Kendall’s tau	13
Spearman’s rank	8
Logistic regression	4
Chi-square	4
ANOVA	2
Chi-square test for trend	1
Comparing percentage of participants with high egg count with percentage of houses they were present in	1
Comparison of percentages	1
Correlation (no further details provided)	1
Fisher’s exact test	1
Looking at association	1
Multiple regression	1
Odds ratio	1
Ranked correlation coefficient	1
Ratio of observed proportion infected and predicted proportion	1
Relative risks	1
Three-level hierarchical statistical model applied to worm counts	1
Transitional probability matrix	1
Variance components analysis	1

^a^Total may exceed 43 due to some papers employing more than one statistical technique

Pre-treatment infection status as a predictor of (re-) infection post-treatment was tested using logistic regression in four papers (9.3%) and multiple regression in one paper (2.3%).

### Evidence for predisposition

Findings will be presented separately for each helminth species. Extended results are summarised in Additional file 2: Table S2.

#### Ascaris lumbricoides

Stronger evidence of predisposition to *A. lumbricoides* infection was found in children than in adults [9–13], and for females as compared to males [10, 12, 14]. It is of note that one study conducted separate analyses for both EPG and expelled worm counts, and evidence for predisposition across all demographic strata was consistently higher from the analysis on expelled worms when compared with those based on epg counts [13]. This is to be expected given the high within- and between-sample variability present in egg counting based on small samples of stool.

Pre-treatment infection intensity as a predictor of post-treatment intensity was commonly investigated for *A. lumbricoides*. All but one of the papers reported a significant correlation between the two time points [15–19], with baseline infection shown to increase odds of re-infection more than two-fold in China [19] and nearly six-fold in Rwanda [17]. However, Krause et al. [20] found no evidence of this correlation in Panama.

Household clustering of *A. lumbricoides* infection was identified in both urban and rural areas in Brazil [21], South Korea [22], Tanzania [23], and Mexico [24]. Additionally, Walker et al. [25] noted that individual predisposition was of limited importance once household clustering of infection had been accounted for. There was evidence of familial predisposition to infection identified in Mexico [11]. However, Chan et al. [26] concluded that any genetic element to predisposition was likely overwhelmed by environmental factors, with the familial predisposition identified in their study being predominantly attributed to parent-to-parent correlations.

#### Trichuris trichiura

Identified studies found strong evidence of predisposition to *T. trichiura* infection in St. Lucia [27], Thailand [28], Malaysia [9] and India [29]. One study in Mexico found stronger predisposition in children than in adults [11]. However, no evidence of predisposition was identified from studies on pre-SAC in Panama [20] or SAC in Jamaica [30].

Household clustering was evident in a study conducted in Mexico, with fewer households observed to be harbouring a single heavily infected person than would be expected if such individuals were randomly distributed throughout the community [24]. Strong evidence of familial predisposition was identified in Mexico [11] but not Malaysia [31].

Baseline *T. trichiura* infection was shown to increase the odds of re-infection by 2.5 times at 4-months post-baseline and 2.3 times 6-months post-baseline, although neither was statistically significant [19]. A significant, positive association between baseline infection intensity and re-infection was found in St. Lucia [32].

#### Hookworm

Strong evidence for predisposition to hookworm infection was consistently found in studies conducted throughout the world. The groups experiencing predisposition, however, appeared to vary by study setting. For example, similar degrees of evidence for predisposition was found in both males and females in Mali [33], whilst studies in India [34] and Papua New Guinea [35] reported that females showed greater evidence of predisposition than males. Studies in India [34] and Zimbabwe [36] suggested that the eldest members of the community showed predisposition to high intensity infection. However, this contrasts with conclusions drawn in both Papua New Guinea [37] and Mali [33], which reported evidence of predisposition amongst both the youngest and oldest individuals. It should be noted that in the case of hookworm the intensity of infection is typically greatest in older rather than younger individuals, which contrasts with both *A. lumbricoides* and *T. trichiura* where the heaviest infection is typically found in SAC [38].

Quinnell et al. [37] found that the increased time between infection status measurements, weakened the evidence for predisposition. However, this finding is contrary to those of Schad & Anderson [7], whose significant evidence of predisposition did not weaken over time.

Two studies conducted in Panama [20, 39] came to contrasting conclusions as to whether pre-treatment infection intensity is a strong predictor of hookworm re-infection, whilst a study conducted in Brazil concluded that a strong relationship between the two measurements did exist [40]. Opposing conclusions were also drawn with regards to household clustering of infection, with significant clustering of heavy hookworm infection identified in Brazil [21, 41], but no evidence of the same pattern was found in Tanzania [23].

#### Schistosomiasis

Children were consistently found to be predisposed to *S. mansoni* infection. Having adjusted for age and sex, strong evidence was found of predisposition amongst Kenyan children aged 1–8 years old and those aged 13–16 [42]. A second study on Kenyan children showed that whilst evidence of predisposition in those aged 9–16 is present up to 21 months post-baseline, it was strongest in the first 12 months after treatment [43]. Results from a study in Brazil, however, found no evidence of predisposition to infection amongst SAC [44]. The intensity of re-infection with *S. mansoni* was found to be associated with the intensity of baseline infection [40]. Evidence of household clustering of *S. mansoni* infection was observed in a Brazilian study, although only in rural areas [21].

Strong evidence of predisposition to *S. haematobium* was identified amongst younger persons in Zimbabwe [45]. Strong evidence of increasing risk of re-infection with increasing pre-treatment infection levels was found in Mali [46]. This was supported by results of a multiple regression analysis which found that, after adjusting for age, school and sex, pre-treatment infection intensity remained a predictor of re-infection amongst SAC in Kenya [47]. Further evidence of high pre-treatment infection intensity being indicative of high post-treatment intensity was identified in Senegalese children, where those with high-intensity baseline infection were almost 11 times more likely to be infected six months post-baseline than those not originally suffering high-intensity infection [48], as well as in Mauritania [49].

Household clustering of *S. japonicum* was identified in the Philippines, with the 38% of study participants with high egg counts residing in just 21% of households [50]. Further evidence of infection clustering within individuals was found in a study conducted in China where the ratio of the observed proportion and predicted proportion of infected individuals was found to be 1.3 and 2.1 in two cohorts with differing infection intensities, both of which were statistically significant results [51]. Of note is that the higher degree of clustering was identified in the cohort with lower infection intensity and prevalence.

## Discussion

The major conclusion from this review is the relative paucity of published epidemiological studies that have investigated the question of why helminth infections are highly aggregated in the human host population and, concomitantly, the linked question of whether those with heavy infection are predisposed to this state. Given the known association of infection intensity (worm burden) with morbidity [52, 53], it would be highly desirable to explore why a few individuals acquire heavy infection and, if predisposed to this state, which factor, or combination of factors (such as social status, behaviour, or host genetics), determine this epidemiological state.

In the current climate of repeated rounds of mass or school-based drug administration, predisposition to heavy infection (or just positive infection) may also be a consequence of poor adherence to treatment over multiple rounds. This leads to a potential definition of predisposition which is completely different to those found in this review. It would also suggest that the definition used for predisposition could be scenario-specific. Predisposition in a study capacity may be defined as consistent re-infection after initial infections have been cleared. Whilst from a control programme point of view, the definition may include (but is not limited to) those that never clear their infections in the first place due to either not taking the drugs, or the drugs being less efficacious on them for some reason. This is an important issue given that persistent non-compliers to treatment may act as a reservoir of infection that hinders efforts to eliminate transmission [54, 55].

In the relatively small number of published papers that address predisposition to either infection (prevalence) or heavy infection (intensity of infection, relative to others in the sampled population), this review has found evidence of predisposition to infection with all species of human helminths for which this epidemiological pattern has been investigated. Children were regularly found to experience greater predisposition to heavy infection with *A. lumbricoides* [9–13], *S. mansoni* [42, 43] and *S. haematobium* [45] than adults, whilst some evidence of the same pattern was identified for *T. trichiura* [11]. Of note is that for these infections, most worms are harboured by children where convex age intensity profiles are the norm [56]. Females were found to be more predisposed to *A. lumbricoides* infection than were males [10, 12, 14]. Household clustering of infection was found to be present for *A. lumbricoides* [21–24], *T. trichiura* [24] and *S. japonicum* [50], with *A. lumbricoides* [11] and *T. trichiura* [11] also showing evidence of familial predisposition. Whilst strong evidence for predisposition to hookworm infection was identified, patterns of which specific groups were affected were considerably more varied than for the other helminth species.

Many papers identified for this review reported similar conclusions to one another with regards to evidence of predisposition. However, findings were not always unanimous. For example, a significant correlation between pre- and post- treatment infection intensity for *A. lumbricoides* was reported by several studies [15–19], whilst Krause et al. [20] found no such pattern. One potential reason for the discrepancy in results could be a combination of the low prevalence post-treatment (9.5%) and the diagnostic tool used; the FLOTAC technique [20]. It has previously been shown that FLOTAC has higher sensitivity for diagnosing infection with *A. lumbricoides* than the Kato-Katz (KK) technique employed by the other papers reporting results on *Ascaris*, especially at low prevalences [57, 58]. This may have resulted in a greater number of infections being diagnosed post-treatment by Krause et al. Furthermore, results presented by Krause et al. [20] show that 21 households were added partway through the study, with no indication given of whether their baseline results differed significantly from households included at the onset of the study. This could explain why the mean intensity of infection was so much higher at the endline that at baseline. Also, the sample size for baseline analysis was 189 whereas for the endline analysis it was 199 [20]. This suggests the two assessments are not being conducted on the exact same people. To get a true idea of predisposition, one must be analysing results of the same individuals at multiple time points, not just individuals from the same community.

Similarly, Bundy et al. [30] found no evidence of predisposition to *T. trichiura* in SAC in Jamaica despite several other studies reporting opposing conclusions, including those by the same authors in St. Lucia [27]. However, Bundy et al.’s study had a small sample size of just 23, as compared to 2098 enrolled by Forrester et al. [24] in Mexico, who did find evidence of predisposition in SAC. The effect of the smaller sample size in Jamaica could be a reduced statistical power for detecting differences between the pre- and post-treatment egg counts, thus resulting in different conclusions to those in other papers.

It is also important to highlight, however, that the cause of opposing results with regards to predisposition could be due to genuine differences in the populations being studied. It has been hypothesised that genetics could play a role in predisposition to helminth infection [7], and it is entirely plausible that different ethnic groups could show differing degrees of predisposition.

Given that sub-Saharan Africa and Asia suffer from the greatest burden of helminth infections [1], our finding that most identified studies were conducted in these regions is logical. Similarly, Fig. 2 shows that all studies were conducted in countries classified as endemic for at least one species of human helminth. Furthermore, the recent increase in publications addressing predisposition, as shown in Fig. 3, coincides with the current increased focus on transmission elimination of human helminths since the 2012 London Declaration.

Conducting a systematic review, and subsequently summarising results from several papers, does not negate any methodological errors present in the individual studies themselves. Some limitations were observed within the research reports included in the review. One important issue is that to determine whether people are consistently being re-infected despite treatment, an assumption must be made that their initial infection was cleared in the first instance. In other words, it is assumed that individuals took the anthelmintic and it acted to clear most of the harboured worms. That said, cure rates recorded for the recommended drugs for treating the human helminths have repeatedly been shown to be high. Albendazole, the leading treatment for hookworm and *A. lumbricoides*, has regularly been shown to cure over 85% of infections [59–62], whist praziquantel has cure rates for schistosomiasis of over 75% [63–66]. However, the cure rates for *T. trichiura* are generally lower, with mebendazole curing between 40 and 70% of infections [61, 62, 67]. As such, the assumption that initial infections were cleared is generally valid, although is perhaps slightly weaker for *T. trichiura*, given the lower cure rates observed in practice.

The methodology employed in this literature review also has limitations. First, only papers published in English were included. With the greatest burden of human helminth infection experienced by those in sub-Saharan Africa and Southeast Asia [1], some papers of relevance not published in English may have been missed. Furthermore, although four different databases were searched (Embase, MEDLINE, Global Health, and Web of Science) other papers relevant to this review may be present in other databases. It seems unlikely, however, that papers missed using the defined methodology will have greatly altered the conclusions drawn in this paper. However, the exclusion of the 52 papers for which a complete text could not be obtained could have an impact on the conclusions drawn in this review.

Another potential limitation of this review is the result of publication bias, whereby only papers deemed to have positive and/or novel findings, are accepted for publication. The near-consistent positive findings with regards to predisposition identified in this review may be due to only these positive papers generally being published, with those finding no evidence of predisposition not deemed worthy of publication. Finally, the search terms used for this review may introduce a further limitation. For example, the lack of papers identified for LF and onchocerciasis may not be due to an absence of relevant studies, but instead due to the search terms not capturing the scientific terminology used when describing these diseases within the literature. As such, a more detailed investigation into predisposition to these two diseases, using an updated and more detailed set of search terms, may be justified.

Aside from the soil-transmitted helminths, other common intestinal nematode infections of humans include strongyloidiasis and enterobiasis. However, neither disease is currently a principal target of either transmission elimination or morbidity control efforts. As such, it was decided that the potential existence of predisposition was not as pressing an issue for these diseases, so they were not included in this review. However, a further review in which predisposition to strongyloidiasis and enterobiasis is investigated could certainly be undertaken.

Whilst it was found that there are groups predisposed to heavy helminth infection, further research is still needed to elucidate specific reasons as to why this is so. Are there particular activities children partake in more often than adults which result in their greater predisposition to *A. lumbricoides*, *S. mansoni* and *S. haematobium*? What part of a women’s daily activities or habits are causing them to experience greater predisposition to *A. lumbricoides*? Although potential reasons are mentioned in papers identified in this review, such as children being more likely to play in ponds, pools, rivers and lakes, therefore increasing their exposure to snails infected with the human schistosome parasites [42], or women defecating in shaded areas where *A. lumbricoides* eggs and hookworm larvae survive longer in the external environment [34], these are only discussed anecdotally. Additionally, behaviour-related aspects of helminth control are most commonly investigated in a cross-sectional manner, at just a single time point [68, 69]. When researching predisposition, however, it is imperative that longitudinal data on these variables are collected such that assessments can be made as to which factors, if any, are affecting the intensity of infection over multiple time points. A more formal approach to identifying risk factors, in which predisposition is the outcome of interest and potential risk factors are assessed by appropriate statistical methods, would allow certain behavioural practices to be highlighted which could then be targeted through educational programs.

Additionally, there was evidence of familial predisposition to *A. lumbricoides* and *T. trichiura* infection, suggesting either a genetic component to predisposition or common behavioural or environmental factors [70]. If genetics is important, further research into gene associations would help shed some light on why certain families are more likely than others to be repeatedly infected, potentially leading to new diagnostic tests aimed at determining likelihood of predisposition [71, 72].

Finally, a consensus on what is meant by predisposition, with a fixed definition and statistical analysis, would not only allow for easier comparisons between studies but would also result in greater clarity when communicating results to program managers in-country. It is important to note that helminth worm burdens are typically highly aggregated in human populations, where most hosts harbour few worms and a few hosts harbour many [56, 73]. The negative binomial probability distribution typically describes observed patterns well. It is defined by two parameters, the mean and a coefficient *k*, which varies inversely with the degree of worm aggregation. The ideal approach to assessing predisposition to heavy or light infection would entail measuring this worm aggregation distribution both pre- and post-treatment, and comparing who lies in the tail with high parasite loads at both time points.

## Conclusion

This review has found evidence of predisposition to high and low worm burdens to human helminth infection, excluding the filarial worms where the issue of predisposition has not been fully explored in the literature yet. This has important implications for the design of MDA treatment programmes in the ‘end game’ when prevalence is very low. Targeted treatment may be best, but cost-benefit studies need to be performed to assess the full benefits of targeted over mass treatment, with the recognition that predisposition to heavy infection may negate the need for repeated measurement of the intensity of infection [74, 75]. Once those predisposed to heavy infection are identified, treatment can continually be targeted to them. It is also clear that little is understood about the factors driving predisposition other than associations with household and family. In the coming years, molecular epidemiological methods based on genome sequencing, perhaps based on single nucleotide polymorphisms (SNPs), could facilitate identifying ‘who infects whom’ and whether certain ethnicities are at higher risk; hence, giving greater insights into the causative factors of predisposition.

## Additional files

Additional file 1: Table S1. PRISMA Checklist for present systematic review. (DOC 67 kb)

Additional file 2: Table S2. Summary of statistical results for all included papers. (XLSX 19 kb)

## Abbreviations

EPG: Eggs per gram
KK: Kato-Katz
LF: Lymphatic filariasis
MDA: Mass drug administration
NGO: Non-governmental organisation
NTDs: Neglected tropical diseases
Pre-SAC: Pre-school-aged children
SAC: School-aged children
SNPs: Single nucleotide polymorphisms
STH: Soil-transmitted helminths
WHO: World Health Organisation
